# High risk of complications following surgical treatment of patella fractures — a cross-sectional study of 798 patients with mean 6.4 years follow-up

**DOI:** 10.1007/s00068-024-02445-7

**Published:** 2024-01-18

**Authors:** Peter Larsen, Marie Arildsen, Kasper Kristensen, Kristian D. Lyng, Rasmus Elsoe

**Affiliations:** 1 Department of Orthopaedic Trauma Surgery, Aalborg University Hospital, Hobrovej 18–22, 9000 Aalborg, Denmark; 2 Department of Physiotherapy, Aalborg University Hospital, Hobrovej 18–22, 9000 Aalborg, Denmark

**Keywords:** Patella, Kneecap, Fracture, Complications, Patient-reported outcome

## Abstract

**Purpose:**

This study aimed to investigate the incidence of early and late complications following treatment of patella fractures. Secondary aims were to investigate the association between early and late complications and the patient-reported outcome measurement, the Knee Injury and Osteoarthritis outcome score (KOOS).

**Methods:**

Cross-sectional study including all patients recorded with a patella fracture residing in the Northern Region of Denmark between 2010 and 2020. Early (before 3 months) and late complications were investigated by retrospective review of charts and x-rays. All patients were invited to participate in the study by reporting current knee-specific symptoms. The KOOS was used to investigate patient-reported knee-specific symptoms.

**Results:**

Seven hundred ninety-eight patients were included in the study. A total of 532 (67%) patients were treated conservatively, and 266 (33%) patients underwent surgery. The mean age at the time of fracture was 66.8, ranging from 6 to 103 years of age. The mean follow-up time was 6.4 years, ranging from 1.1 to 12.3 years follow-up. Overall, the rate of complications was 26%. Overall, the rate of complication for the surgical group was 57% and for the conservative group 4%. The most common early complication was the loss of reduction followed by the removal of symptomatic hardware. The most common late complication was the removal of symptomatic hardware and knee arthroscopy. In all the five KOOS subscales (Pain, Symptoms, ADL, Sport/Rec, and QOL), patients presenting with early and late complications reported statistically significantly worse scores than those without complications.

**Conclusion:**

The overall incidence of complications in patients presenting with a patella fracture was 26%, with a mean follow-up time of 6.4 years. In the surgical group, 57% of patients experience at least one complication during the follow-up period. Early and late complications were significantly associated with worse KOOS subscale  scores.

## Introduction

Patella fractures are frequent in orthopaedic emergency departments, with an overall incidence of 13.1/100,000/year [1]. The mode of injury is reported with a bimodal distribution with older women caused by falls and younger men caused by falls from height, road traffic accidents, and sport [1].

The aim when treating patella fractures is to restore the knee extensor mechanism and the patella articular surface [2]. Both surgical and conservative management are used, depending on fracture classification, fracture dislocation, and impairment of the extensor mechanism [2–5].

The current standard surgical procedure includes tension band wiring for transverse and comminuted fractures [2, 6–9]. Screws and Kirschner wires are commonly used with tension band wiring [2, 6–9]. Moreover, plate fixation of patella fractures has become increasingly popular in recent years [10, 11]. Conservative treatment included immobilisation of the knee joint in a cast or brace for 6–8 weeks [2].

Early and long-term complications following patella fractures are common [6, 12–20]. Early complications include implant failure and loosening, deep or superficial infection, deep vein thrombosis, and tendon/wound rupture [12]. Late complications include symptomatic hardware, knee stiffness, prolonged knee pain, loss of muscle strength, decreased quality of life, restriction in knee function, non-union, and posttraumatic osteoarthritis [12]. The overall reoperation rate following surgical treatment of patella fractures has been reported to be 34% [12]. Increasing co-morbidity is reported to increase the risk of postoperative complications [21, 22].

Although a high rate of both early and late complications following surgical treatment of patella fractures is common, current literature lacks large-scale clinical studies investigating the incidence of complications [12]. A meta-analysis with the aim to investigate the frequency of complications in surgical treated patella fractures by Dy et al. [12] reported 33.6% reoperation, 3.3% infection, and 1.3% non-union. The impact of early and late complications on patient-reported outcomes lacks evidence.

The present study aimed to investigate the incidence of early and late complications following treatment of patella fractures.

Secondary aims were to investigate the association between early and late complications and the patient-reported outcome measurement, the Knee Injury and Osteoarthritis outcome score (KOOS).

The hypothesis was that surgical treatments of patella fractures were associated to a considerable risk of complications. Moreover, the hypothesis was that patients with complications would report worse KOOS scores compared to patients without complications.

## Methods and materials

### Study design

Cross-sectional data were collected by retrospective charts and x-rays review and by the prospective patient-reported outcome. The primary outcome was the incidence of pre-defined early and late complications.

The Danish Data Protection Agency (journal number, 2021–218) and the Committee for Science of Northern Denmark (journal number, 21102021) approved the study, which was performed according to the principles of the Helsinki Declaration. The reporting of the study complies with the Strengthening the Reporting of Observational Studies in Epidemiology (STROBE) statement [23].

### Recruitment of patients with patella fractures

All patients recorded with a patella fracture and residing in the Northern Region of Denmark between 2010 and 2020 were included in the study. The defined area of Northern Denmark includes an average population of 584,991 during the study period. The treatment of patella fractures in the Northern Region of Denmark is served by Aalborg University Hospital, a level 1 Trauma Centre, and two smaller regional hospitals.

**Fig. 1:**
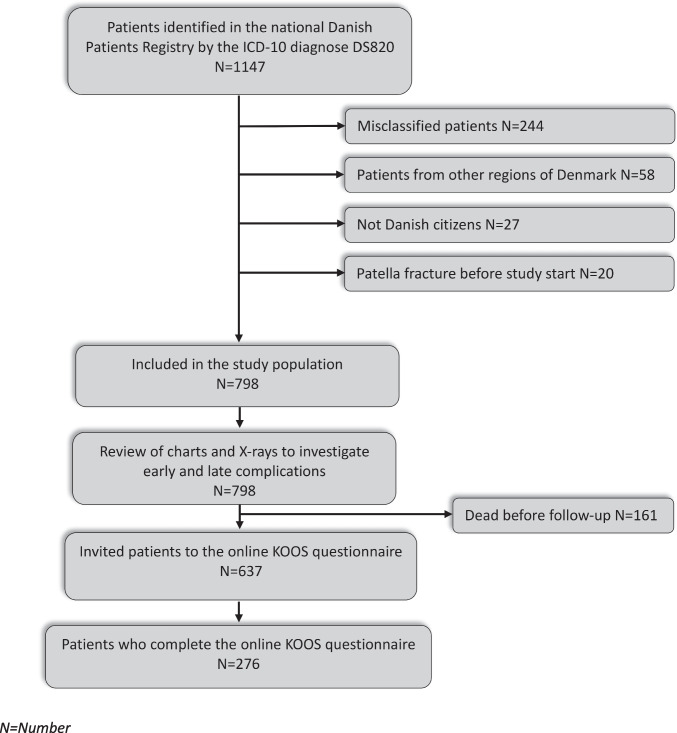
Detailed flow of the study

Patients were identified in the national Danish Patients Registry by the ICD-10 diagnosis DS820 [24]. All contacts with the health care system in Denmark are registered digitally in the Danish Patient Registry as required by Danish law and are linked to a unique ID number (CPR) identifying the person [25]. Excluded were patients from other regions of Denmark and patients who were not Danish citizens. Moreover, patients with patella fractures prior to 2010 were excluded. Moreover, misclassified patients were excluded following a review of charts and x-rays (Fig. 1).

### Outcomes

Baseline outcomes were obtained from charts and x-rays review. Baseline data constitutes age at the time of fracture, follow-up time, sex, mode of injury, AO fracture classification [26, 27] (evaluated by pre-operative x-rays or, if available, computer tomography scan (CT)), conservative or surgical treatment, and, if relevant, type of surgical procedure.

#### Primary outcome

Early and late complications were investigated by retrospective review of charts and x-rays.

Early complications were pre-defined as implant failure and loosening, deep or superficial infection, deep vein thrombosis, tendon/wound rupture, and secondary surgery within the first 3 months following primary treatment.

Late complications were pre-defined as symptomatic hardware, knee stiffness, prolonged knee pain, non-union, reporting of posttraumatic osteoarthritis, and secondary surgery after 3 months following primary treatment, including all procedures related to the patella fracture and secondary treatment with total knee replacement (TKR) or knee arthroscopy.

#### Secondary outcome

All patients were invited to participate in the study by reporting current knee-specific symptoms. The KOOS was used to investigate patient-reported knee-specific symptoms [28]. All patients receive an invitation letter, including an online link to the KOOS and contact information for the research group in case of questions. If there was no response, a second request was forwarded after 14 days.

The KOOS is a patient-reported and knee-specific questionnaire that includes five subscales: pain, symptoms, daily living function (ADL), sport and recreation function (Sport/Rec), and quality of life (QOL) [28]. The score of each subscale is calculated based on standardised scoring algorithms and presented as a score between 0 and 100 [28]. A score of 100 indicates the best possible results and 0 the worst outcome [28].

### Statistics

Continuous data were expressed as mean and standard deviation (SD). Categorical data were expressed as frequencies. An ANOVA test was used to analyse the difference between KOOS subscales and early/late/no complications. If a significant ANOVA factor was found, multiple pairwise analyses with post hoc test (Bonferroni) corrections were used.

A *P*-value of < 0.05 was considered significant. All analyses were performed using Stata statistical software (StataCorp LP).

## Results

A total of 1147 patients were identified. Following a manual review of all individual charts and x-rays, a total of 798 patients were included in the study population. A total of 532 (67%) patients were treated conservatively, and 266 (33%) patients underwent surgery. A detailed flow of the study population is presented in Fig. 1.

The mean age at the time of fracture was 66.8, with a range from 6 to 103 years of age. Women represent 63% and men 37% of the study population. The mean follow-up time was 6.4 years, with a range from 1.1 to 12.3 years follow-up. Detailed baseline characteristics of the study population are outlined in Table 1.

**Table 1:** Baseline characteristics

Baseline characteristics	Total
Number of patients	798
Age at the time of fracture (mean, sd)	66.8 (20.1)
Sex women, %, *n*	63% (502)
Follow-up time	6.4 (3.3)
Fracture classification, %, *n*	
34-A	20% (163)
34-B	16% (130)
34-C	63% (505)
Side of fracture left/right, % (*N*)	44% (352)/56% (446)

### Primary outcome

#### Incidence of early and late complications

Overall, the rate of complications was 26% (212 complications in 171 patients) during the full follow-up procedure. Early complications were 74 in 63 patients, and late complications were 138 in 121 patients. Thirteen patients experience both early and late complications. At the time of fracture, the mean ages of patients presenting with early, late, and no complications were 71.3, 61.2, and 66.2 years, respectively.

The most common early complication was the loss of reduction followed by the removal of symptomatic hardware. The most common late complication was the removal of symptomatic hardware and knee arthroscopy. A detailed overview of all identified complications divided by total and treatment groups is presented in Table 2 and 3.

**Table 2:** Characteristics of the 266 patients treated surgically and the 532 patients treated conservatively

Surgical treatment			
	34 A	34 B	34 C
Number of patients	39	5	222
Age at the time of fracture (mean, sd)	57.6 (23.1)	64.7 (21.7)	67.3 (18.0)
Sex women, %, *n*	56% (22)	60% (3)	62% (138)
Follow-up time	7.4 (3.6)	6.0 (4.7)	6.4 (3.1)
Surgical procedure, *N*			
K-wire	11	2	164
Cerclage/suture/TBW	27	2	199
Screws	12	3	43
Plate	0	0	13
Patients with early complications, *N*	3	2	58
Patients with late complications, *N*	24	3	94
Conservative treatment			
	34 A	34 B	34 C
Number of patients	124	125	282
Age at the time of fracture (mean, sd)	58.3 (23.9)	65.8 (17.9)	72.0 (18.8)
Sex women, %, *n*	54% (67)	53% (66)	73% (205)
Follow-up time	6.9 (3.6)	6.0 (3.3)	6.3 (3.2)
Patients with early complications, *N*	0	2	2
Patients with late complications, *N*	6	2	7

**Table 3:** Early and late complications for the total sample and for the surgical group

Complications	Total sample *N* = 798		Surgical *N* = 266	
Number of patients with early complications, *N*	63	8%	59	22%
Early complications prevalence, *n*, %	74	9%	69	26%
Implant failure and loosening	41	5%	41	15%
Deep infection	10	1%	10	4%
Superficial infection	10	1%	10	4%
Wound rupture	1	< 1%	1	< 1%
Symptomatic hardware removal before 3 months	6	1%	6	2%
Q-tendon rupture	1	< 1%	1	< 1%
Other	5	1%	1	< 1%
Number of patients with late complications, *N*	121	15%	106	40%
Late complications prevalence, *N*, %	138	17%	119	45%
Knee arthroscopy	27	3%	16	6%
Total knee replacement	6	0.8%	0	0
Symptomatic hardware	103	13%	103	39%
Other	2	< 1%	0	0

Overall, the complication rate for the surgical group was 188 in 152 patients, indicating that 57% of the patients in the surgical group experienced at least one complication. The rate of complication for the conservative group was 19 in 19 patients, indicating that 4% of patients in the conservative group experienced a complication. Characteristics of the surgical and the conservative groups divided by fracture classification are presented in Table 2.

### Secondary outcomes

#### Characteristics of patients presenting with loss of reduction before union

Further descriptive information of the subgroup of patients presenting with the early loss of reduction (*N* = 41) showed a mean age of 75.5 years, and the AO classification included only type C fractures.

#### Patient-reported outcome measurement

A total of 276/637 (43%) patients completed the online KOOS questionnaire (at the time of follow-up, a total of 161 were deceased). The mean follow-up time of patients who completed the KOOS questionnaire was 5.9 years with a range from 1.3 to 12.2 years. Of the 276 patients who completed the KOOS questionnaire, 111 (40%) patients underwent ORIF.

The KOOS outcomes divided by subscale scores (Pain, Symptoms, ADL, Sport, and QOL) in patients presenting with early and late complications and in patients without complications are presented in Table 4. The KOOS scores divided by fracture classification and in conservative or surgical treatments are presented in Table 5.

**Table 4:** KOOS subscale scores

KOOS	Pain	Symptoms	ADL	Sport/Rec	QOL
All patients = 276					
Mean, 95%CI	80.3 (77.8–82.7)	79.0 (76.6–81.3)	80.0 (77.5–82.4)	56.5 (52.7–60.3)	64.7 (61.4–67.9)
Early complications = 18					
Mean, 95%CI	68.9 (53.8–84.0)	68.8 (57.6–80.0)	60.8 (47.7–73.9)	29.3 (12.9–45.7)	38.8 (24.2–53.3)
Late complications = 53					
Mean, 95%CI	70.1 (63.3–76.9)	68.2 (61.8–74.6)	71.9 (65.4–78.4)	41.7 (33.2–50.2)	52.4 (44.6–60.1)
No complications = 205					
Mean, 95%CI	83.6 (81.1–86.0)	82.3 (79.9–84.7)	83.4 (80.8–85.9)	62.3 (58.1–66.5)	69.7 (66.2–73.3)

**Table 5:** KOOS subscale scores divided by treatment and fracture classification

KOOS	Pain	Symptoms	ADL	Sport/Rec	QOL
Surgical, mean, 95%CI					
34A, *N* = 17	79.7 (68.8–90.7)	77.3 (67.3–87.3)	82.2 (73.4–91.0)	55.0 (41.4–68.6)	60.7 (48.0–73.3)
34B, *N* = 1	na	na	na	na	na
34C, *N* = 90	77.7 (73.4–82.1)	74.4 (70.4–78.4)	76.5 (72.4–80.7)	44.9 (38.4–51.4)	57.7 (52.2–63.1)
Conservative, mean, 95%CI					
34A, *N* = 45	81.3 (75.4–87.2)	81.3 (75.3–87.3)	81.1 (74.6–87.7)	62.8 (52.1–73.4)	68.5 (59.3–77.7)
34B, *N* = 48	81.7 (75.0–88.5)	82.9 (77.1–88.7)	80.5 (73.7–87.2)	58.7 (49.6–67.7)	68.5 (60.2–76.8)
34C, *N* = 75	81.3 (77.0–85.7)	80.5 (76.0–85.0)	82.1 (77.6–86.6)	64.9 (58.0–71.8)	68.8 (62.4–75.1)

Results showed that both early and late complications might result in significantly worse KOOS outcomes. In all the five KOOS subscales (Pain, Symptoms, ADL, Sport/Rec, and QOL), patients presenting with early complications reported statistically significantly worse scores than those without complications. Pain (mean difference 14.9 95%CI 1.4–28.4), Symptoms (mean difference 13.2 95%CI 0.3–26.1), ADL (mean difference 22.7 95%CI 8.8–36.5), Sport/Rec (mean difference 32.9, 95%CI 11.3–54.6), and QOL (mean difference 31.9, 95%CI 13.7–50.1).

Moreover, patients presenting with late complications reported statistically significantly worse scores compared to patients without complications for all five KOOS subscales. Pain (mean difference 13.5, 95%CI 5.5–21.5), Symptoms (mean difference 14.2, 95%CI 6.6–21.8), ADL (mean difference 11.6, 95%CI 3.5–19.5), Sport/Rec (mean difference 20.6, 95%CI 8.1–33.1), and QOL (mean difference 17.1, 95%CI 6.3–27.8).

## Discussion

This study investigated the incidence of complications in 798 consecutive patients presenting with a patella fracture treated either conservatively (67%) or surgically (33%). The overall complication rate was 26% with a mean follow-up time of 6.4 years. We demonstrated that both early (9%) and late complications (17%) following patella fractures are common. Eight out of every nine (89%) complications were observed in the surgical group. In the surgical group, 57% of patients experience at least one complication during the follow-up period. Early and late complications were significantly associated with worse KOOS subscale scores.

### Early and late complications are incident following patella fractures

The complication rate following patella fractures is substantial (26%); however, serious complications such as deep infection and implant failure/loosening are rare. Among surgically treated patients, 57% of patients experienced at least one complication in the mean 6.4 years observational period. Among conservatively treated patients, 4% experienced a complication in the mean 6.4 years observational period. This information should be included when informing patients of the expectations of treatment, outcome, additional need for surgery, and recovery.

Symptomatic hardware removal was the most common late complication experienced by 39% of patients in our study. Results are in line with existing literature reporting high rates of symptomatic hardware following surgical treatment of patella fractures [12, 13, 20, 29–31]. Direct comparison of results to the existing literature is difficult due to differences in surgical fixation methods, limited follow-up time, and/or smaller case series. The mean age of patients presenting with late complications was 61.2 years, indicating that symptomatic hardware is most pronounced in the younger and more active age groups. The authors speculate that physical activity may be related to patient-perceived symptomatic hardware. More research is needed to investigate this speculative hypothesis.

The most common early complication was the loss of reduction, requiring additional surgery (15%). Results are almost similar to what was reported from other studies reporting on early complications to the surgical treatment of patella fractures [32, 33]. Loss of reduction has been associated with older age, the use of K-wires in combination with TBW, and biomechanical properties of the surgical technique [32, 33]. Patients in this study presenting with an early loss of reduction were all treated with the modified TBW technique, including the double knot. The authors speculate that fracture complexity in combination with osteoporotic bone may influence the risk of early loss of reduction as the mean age of patients was 8 years older than the overall average and were solely type C fractures. This information may indicate that the standard surgical procedure with tension band wiring may be less successful in comminuted fractures sustained by older patients. The development and introduction of plate fixation to comminuted patella fractures have, in recent years, been more common, and recent literature supports a lower rate of complications compared to tension band wiring, although no large-scale studies or RCT studies are available [10, 11, 34].

### Patient-report outcome measurement, KOOS

One of the most important findings from this study is that early and late complications were significantly associated with worse KOOS subscale cores. A substantial mean difference in the KOOS subscale scores between patients with and without complications was observed among all KOOS subscales and most pronounced on the subscales Sport/Rec and QOL, with more than 17 points in difference between patients presenting with and without complications.

With clinically important differences in all subscales (MCID of about 10 points [28]), the high rate of complication following surgically treated patella fractures represents an important challenge and an area for improvement in the quality of care of patients. Furthermore, results indicated that early complications such as implant failure and deep/superficial infections during the first 3 months after treatment significantly influence the worse KOOS subscale scores. To the authors’ knowledge, no real advancement in the surgical treatment of patella fractures has been achieved in recent decades, although the rate of both early and late complications seems persistently high. Data may change when more evidence on plate fixation becomes available.

### Strength and limitations

The major strength of this study is the large sample of 798 consecutive patients presenting with a patella fracture evaluated manually by chart and x-rays review to explore the risk of complications. Another strength is data regarding the association between early and late complications and the patient-reported outcome, which is seldom discussed in the existing literature.

A limitation may be the 43% of patients responding to the KOOS questionnaire. An analysis between responders and non-responders did not differ in age and gender. Moreover, the age of the patients is very wide (6 to 103) which may cause variation in the results regarding the need for surgical treatment and the rate of complications especially the loss of reduction. Another limitation of the study may be that few patients have not been included in the study population due to an error in primary diagnosis coding to the Danish Patients Registry. However, reporting to the Danish Patients Registry is required by law in Denmark, and the positive predictive values of orthopaedic diagnoses in the Danish Patients Registry are reported to be high [35].

## Conclusion

This study investigated the incidence of complications in 798 consecutive patients presenting with a patella fracture treated conservatively (67%) or surgical (33%). The overall complication rate was 26% with a mean follow-up time of 6.4 years. In the surgical group, 57% of patients experienced at least one complication during the follow-up period. Among conservatively treated patients, 4% experienced a complication in the mean 6.4 years observational period. Early and late complications were significantly associated with worse KOOS subscale scores.

Clinical practice should use this information to guide patients’ expectations regarding treatment, outcome, additional surgery, and recovery needs. Furthermore, the high rates of complication following surgical treatment of patella fractures represent an important challenge in the overall care of patients experiencing a patella fracture.

## Data Availability

Data is available on request.
